# Association between Respiratory Syncytial Virus Activity and Pneumococcal Disease in Infants: A Time Series Analysis of US Hospitalization Data

**DOI:** 10.1371/journal.pmed.1001776

**Published:** 2015-01-06

**Authors:** Daniel M. Weinberger, Keith P. Klugman, Claudia A. Steiner, Lone Simonsen, Cécile Viboud

**Affiliations:** 1 Department of Epidemiology of Microbial Diseases, Yale School of Public Health, New Haven, Connecticut, United States of America; 2 Division of International Epidemiology and Population Studies, Fogarty International Center, National Institutes of Health, Bethesda, Maryland, United States of America; 3 Department of Global Health, Rollins School of Public Health, Emory University, Atlanta, Georgia, United States of America; 4 Healthcare Cost and Utilization Project, Agency for Healthcare Research and Quality, Rockville, Maryland, United States of America; 5 Department of Global Health, George Washington University, Washington, District of Columbia, United States of America; University of Oxford, Thailand

## Abstract

**Background:**

The importance of bacterial infections following respiratory syncytial virus (RSV) remains unclear. We evaluated whether variations in RSV epidemic timing and magnitude are associated with variations in pneumococcal disease epidemics and whether changes in pneumococcal disease following the introduction of seven-valent pneumococcal conjugate vaccine (PCV7) were associated with changes in the rate of hospitalizations coded as RSV.

**Methods and Findings:**

We used data from the State Inpatient Databases (Agency for Healthcare Research and Quality), including >700,000 RSV hospitalizations and >16,000 pneumococcal pneumonia hospitalizations in 36 states (1992/1993–2008/2009). Harmonic regression was used to estimate the timing of the average seasonal peak of RSV, pneumococcal pneumonia, and pneumococcal septicemia. We then estimated the association between the incidence of pneumococcal disease in children and the activity of RSV and influenza (where there is a well-established association) using Poisson regression models that controlled for shared seasonal variations. Finally, we estimated changes in the rate of hospitalizations coded as RSV following the introduction of PCV7. RSV and pneumococcal pneumonia shared a distinctive spatiotemporal pattern (correlation of peak timing: ρ = 0.70, 95% CI: 0.45, 0.84). RSV was associated with a significant increase in the incidence of pneumococcal pneumonia in children aged <1 y (attributable percent [AP]: 20.3%, 95% CI: 17.4%, 25.1%) and among children aged 1–2 y (AP: 10.1%, 95% CI: 7.6%, 13.9%). Influenza was also associated with an increase in pneumococcal pneumonia among children aged 1–2 y (AP: 3.2%, 95% CI: 1.7%, 4.7%). Finally, we observed a significant decline in RSV-coded hospitalizations in children aged <1 y following PCV7 introduction (−18.0%, 95% CI: −22.6%, −13.1%, for 2004/2005–2008/2009 versus 1997/1998–1999/2000). This study used aggregated hospitalization data, and studies with individual-level, laboratory-confirmed data could help to confirm these findings.

**Conclusions:**

These analyses provide evidence for an interaction between RSV and pneumococcal pneumonia. Future work should evaluate whether treatment for secondary bacterial infections could be considered for pneumonia cases even if a child tests positive for RSV.

*Please see later in the article for the Editors' Summary*

## Abstract 2

Daniel Weinberger and colleagues examine a possible interaction between two serious respiratory infections in children under 2 years of age.

*Please see later in the article for the Editors' Summary*

## Editors' Summary

**Background:**

Respiratory infections—bacterial and viral infections of the lungs and the airways (the tubes that take oxygen-rich air to the lungs)—are major causes of illness and death in children worldwide. Pneumonia (infection of the lungs) alone is responsible for about 15% of all child deaths. The leading cause of bacterial pneumonia in children is *Streptococcus pneumoniae*, which is transmitted through contact with infected respiratory secretions. *S. pneumoniae* usually causes noninvasive diseases such as bronchitis, but sometimes the bacteria invade the lungs, the bloodstream, or the covering of the brain, where they cause pneumonia, septicemia, or meningitis, respectively. These potentially fatal invasive pneumococcal diseases can be treated with antibiotics but can also be prevented by vaccination with pneumococcal conjugate vaccines such as PCV7. The leading cause of viral pneumonia is respiratory syncytial virus (RSV), which is also readily transmitted through contact with infected respiratory secretions. Almost all children have an RSV infection before their second birthday—RSV usually causes a mild cold-like illness. However, some children infected with RSV develop pneumonia and have to be admitted to hospital for supportive care such as the provision of supplemental oxygen; there is no specific treatment for RSV infection.

**Why Was This Study Done?:**

Co-infections with bacteria and viruses can sometimes have a synergistic effect and lead to more severe disease than an infection with either type of pathogen (disease-causing organism) alone. For example, influenza infections increase the risk of invasive pneumococcal disease. But does pneumococcal disease also interact with RSV infection? It is important to understand the interaction between pneumococcal disease and RSV to improve the treatment of respiratory infections in young children, but the importance of bacterial infections following RSV infection is currently unclear. Here, the researchers undertake a time series analysis of US hospitalization data to investigate the association between RSV activity and pneumococcal disease in infants. Time series analysis uses statistical methods to analyze data collected at successive, evenly spaced time points.

**What Did the Researchers Do and Find?:**

For their analysis, the researchers used data collected between 1992/1993 and 2008/2009 by the State Inpatient Databases on more than 700,000 hospitalizations for RSV and more than 16,000 hospitalizations for pneumococcal pneumonia or septicemia among children under two years old in 36 US states. Using a statistical technique called harmonic regression to measure seasonal variations in disease incidence (the rate of occurrence of new cases of a disease), the researchers show that RSV and pneumococcal pneumonia shared a distinctive spatiotemporal pattern over the study period. Next, using Poisson regression models (another type of statistical analysis), they show that RSV was associated with significant increases (increases unlikely to have happened by chance) in the incidence of pneumococcal disease. Among children under one year old, 20.3% of pneumococcal pneumonia cases were associated with RSV activity; among children 1–2 years old, 10.1% of pneumococcal pneumonia cases were associated with RSV activity. Finally, the researchers report that following the introduction of routine vaccination in the US against *S. pneumoniae* with PCV7 in 2000, there was a significant decline in hospitalizations for RSV among children under one year old.

**What Do These Findings Mean?:**

These findings provide evidence for an interaction between RSV and pneumococcal pneumonia and indicate that RSV is associated with increases in the incidence of pneumococcal pneumonia, particularly in young infants. Notably, the finding that RSV hospitalizations declined after the introduction of routine pneumococcal vaccination suggests that some RSV hospitalizations may have a joint viral–bacterial etiology (cause), although it is possible that PCV7 vaccination reduced the diagnosis of RSV because fewer children were hospitalized with pneumococcal disease and subsequently tested for RSV. Because this is an ecological study (an observational investigation that looks at risk factors and outcomes in temporally and geographically defined populations), these findings do not provide evidence for a causal link between hospitalizations for RSV and pneumococcal pneumonia. The similar spatiotemporal patterns for the two infections might reflect another unknown factor shared by the children who were hospitalized for RSV or pneumococcal pneumonia. Moreover, because pooled hospitalization discharge data were used in this study, these results need to be confirmed through analysis of individual-level, laboratory-confirmed data. Importantly, however, these findings support the initiation of studies to determine whether treatment for bacterial infections should be considered for children with pneumonia even if they have tested positive for RSV.

**Additional Information:**

Please access these websites via the online version of this summary at http://dx.doi.org/10.1371/journal.pmed.1001776.

- The US National Heart, Lung, and Blood Institute provides information about the respiratory system and about pneumonia
- The US Centers for Disease Control and Prevention provides information on all aspects of pneumococcal disease and pneumococcal vaccination, including personal stories and information about RSV infection
- The UK National Health Service Choices website provides information about pneumonia (including a personal story) and about pneumococcal diseases
- KidsHealth, a website provided by the US-based non-profit Nemours Foundation, includes information on pneumonia and on RSV (in English and Spanish)
- MedlinePlus provides links to other resources about pneumonia, RSV infections, and pneumococcal infections (in English and Spanish)
- HCUPnet provides aggregated hospitalization data from the State Inpatient Databases used in this study

## Introduction

Respiratory infections caused by viruses and bacteria account for a substantial burden of disease in children throughout the world. Co-infections of bacteria and viruses can have a synergistic effect and lead to more severe disease and hospitalization. In order to design and implement more effective interventions against respiratory infections, it is critical to better understand these interactions. The classic example of this type of interaction is between pneumococcus and influenza. Influenza infections increase the risk for pneumococcal disease during both pandemic and inter-pandemic periods [1]–[8]. However, there is some evidence that pneumococcus also interacts with respiratory syncytial virus (RSV), which causes a large proportion of respiratory infections in young children [9]. Children hospitalized with RSV in Denmark had an elevated risk for developing invasive pneumococcal disease during the following month [10], and time series analyses that controlled for seasonality found an association between RSV activity and invasive pneumococcal disease [11],[12]. Additionally, a randomized controlled trial of the pneumococcal vaccine in South Africa found that HIV-negative vaccine recipients had a 32% lower risk for hospitalization with proven RSV-associated pneumonia than unvaccinated children [13]. Finally, sequential infections of mice with RSV and pneumococcus demonstrate that the primary viral infection decreases bacterial clearance from the lung [14] and increases bacterial virulence [15]. These observations of an association between pneumococcus and RSV seemingly contradict clinical findings that bacteremia rarely occurs in children hospitalized with RSV [16], as well as a recent study from England and Wales that found no association between RSV and invasive pneumococcal disease in children aged <5 y [17].

Quantifying the relationship between RSV and pneumococcal infections can be difficult because of the fact that rates of disease caused by both pathogens increase during winter months. The interaction could be evaluated in the context of a randomized controlled vaccine trial (as was done in South Africa [13]), in studies that evaluate the prevalence of the virus and bacteria among cases and controls, and in analyses of seasonal [4] and long-term trends at the population level. Numerous studies have identified a correlation between pneumococcal incidence and RSV activity, but these studies can be difficult to interpret because both pathogens have seasonal patterns and likely share other unexplained risk factors [4],[18]–[21]. However, such ecological studies can be informative if the analyses control for seasonality using either weather variables [12],[17] or other variables that change seasonally [11],[22]. To convincingly establish a relationship between these two pathogens, one must demonstrate that they share similar spatiotemporal epidemic patterns across diverse geographical locations and that interventions that target one of the pathogens influence the incidence of the other.

In this study, we explored the relationship between RSV and pneumococcal disease using data from a large multi-state hospitalization database from the United States. We considered whether RSV and pneumococcal pneumonia share a similar spatiotemporal pattern across the US. We then quantified the association between RSV and pneumococcal pneumonia and pneumococcal septicemia while controlling for shared seasonal variations and influenza activity using harmonic regression. Finally, we evaluated whether pediatric vaccination with seven-valent pneumococcal conjugate vaccine (PCV7) was associated with a decline in the incidence of hospitalizations coded as RSV. We discuss how these findings could help to resolve the apparent contradictions among previous studies of pneumococcus–RSV co-infection.

## Methods

### Data Sources and Extraction

Weekly hospitalization data were obtained from the State Inpatient Databases of the Healthcare Cost and Utilization Project, maintained by the Agency for Healthcare Research and Quality, through an active collaboration. These database contains all hospital discharge records from community hospitals in participating states [23]. For these analyses, data were available from 1992/1993 to 2008/2009. Data were not available for all states for all years (Figure S1). The pneumococcal vaccine was introduced in the United States in 2000. For analyses of post-PCV7 changes in incidence, we used data from the 18 states that had at least two seasons of pre-PCV7 data and three seasons of post-PCV7 data. For the analyses of epidemic timing, we estimated timing using data only from states that had an average of at least ten hospitalizations per year for the outcome being evaluated. Data about population sizes for each state were obtained from Census Bureau statistics compiled by the Surveillance, Epidemiology, and End Results Program [24]. All analyses were performed in SAS version 9.2 (SAS Institute, Cary, North Carolina).

Cases were identified by the presence of the relevant diagnostic discharge codes listed anywhere in the patient's discharge record, including pneumococcal pneumonia/lobar pneumonia (International Classification of Diseases, Ninth Revision [ICD-9], code 481), pneumococcal septicemia (038.2), respiratory syncytial virus (079.6, 466.11, 480.1), influenza (487.0–487.9), and bronchiolitis (466). Cases were aggregated by week, disease outcome, state, and age stratum (0–11 mo, 12–23 mo). We further subdivided the < 1-y-old age group into the groups 0–2 mo and 3–11 mo because serotype replacement following pneumococcal vaccination has been more severe among neonates than among older children [25]. The variable used to create the age strata 0–2 mo and 3–11 mo was available for fewer states (Arizona, California, Colorado, Georgia, Iowa, Kansas, Massachusetts, New York, South Carolina) than the variable used to create the age strata 0–11 mo and 12–23 mo (Figure S1). When fitting the regression models containing influenza and RSV as covariates, we used the total viral incidence across all age categories to minimize potential biases related to testing in specific age strata. As a sensitivity analysis for the RSV coding, we repeated the regression but substituted weekly bronchiolitis among < 1-y-old children for the RSV variable.

### Harmonic Regression to Estimate Epidemic Timing

We used regression models to estimate the average peak timing of RSV, pneumococcal pneumonia, and pneumococcal septicemia among < 2-y-old children by state. The outcome variable was disease incidence in each state, as measured by hospitalizations, and the predictors were sine and cosine terms with periods of 52.25 and 104.5 wk. These harmonic terms capture both annual and biennial epidemic patterns [26]. Data were included from 1992/1993 to 2008/2009, as available (Figure S1). In order to adjust for changes in overall incidence caused by vaccination or coding changes, an offset term was included that was equal to the average incidence for the corresponding 12-mo July–June period. The model was
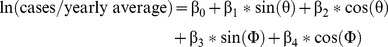
(1)where θ = 2_*_π_*_week/52.25 and Ф = 2_*_π_*_week/104.5, representing 1-y and 2-y epidemic cycles, respectively. The timing of the epidemic peak (phase angle) and the confidence intervals were calculated from the regression coefficients of the 1-y sine and cosine terms (β_1_ and β_2_), as described in [26], and then converted from radians to weeks [26]. The scale was shifted so that the minimum value (RSV in Florida) was week 1. The timing of RSV epidemics was compared with the timing of pneumococcal pneumonia or septicemia epidemics using Pearson's correlations. Poisson regression was used to fit the model to the pneumococcal pneumonia and pneumococcal septicemia time series. Because the RSV data exhibited evidence of overdispersion, we used negative binomial regression, rather than Poisson regression, to fit the model to the RSV time series. The choice of using Poisson or negative binomial regression influences the standard errors of the estimates but would not be expected to influence the point estimates. The regressions were fit separately for each state and outcome.

### Regression of RSV on Pneumococcal Pneumonia

To quantify the association of RSV and influenza activity with pneumococcal disease incidence while controlling for seasonal variations, we fit a Poisson regression model. This model includes harmonic variables that cycle every 6 and 12 mo. These variables capture seasonal fluctuations that are consistent between years and allow for the comparison of weekly pneumococcal disease rates and RSV and influenza rates while subtracting out the consistent seasonal variations. For this analysis, we focused on the period from 1997/1998 to 2008/2009, which excludes the period prior to when the ICD-9 codes for RSV changed, and it excludes the second half of 2009 (which had unreliable influenza coding due to the 2009 pandemic) and the start of the 13-valent pneumococcal conjugate vaccine (PCV13) period. The outcome variable was weekly incidence of pneumococcal pneumonia or pneumococcal septicemia hospitalizations. The Bayesian information criterion (BIC) was used to identify the best model. This criterion provides an estimate of the goodness of fit of the model while including a penalty for overfitting (including too many variables). The model, based on Simonsen at al. [27], was
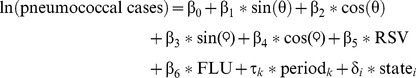
(2)where θ = 2_*_π_*_week/52.25 and 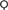 = 2_*_π_*_week/26.125. The sine and cosine terms fit a seasonal baseline using annual and semi-annual periods. δ*_i_* estimates the state-specific intercept along with β_0_. RSV and FLU are the weekly incidence of RSV and influenza hospitalizations, respectively, in all ages in each state. τ*_k_* estimates the change in incidence between the pre-vaccine period (1997/1998–1999/2000), early post-vaccine period (2000/2001–2002/2003), and late post-vaccine period (2003/2004–2008/2009). We also evaluated simpler and more complex models that included interaction terms that allowed the seasonal and viral variables to vary by state. Based on BIC, the model presented above provided the best fit. Estimates from the alternative models are presented in Text S1, Table S1, and Figure S4.

The percent of pneumococcal disease hospitalizations attributable to influenza or to RSV were calculated based on the model estimates [8],[22]: we obtained an estimate of the predicted incidence of pneumococcal disease hospitalizations in each week using the equation above, inserting estimates for each of the parameters along with the observed weekly values of influenza and RSV hospitalization rates. To obtain an estimate of the expected incidence of pneumococcal disease if influenza or RSV was not present, we used this same equation (without refitting the model) and set the influenza or RSV term equal to zero. The overall predicted incidence and the predicted incidence with the incidence of influenza or RSV set to zero were then summed across all weeks. The incidence of pneumococcal disease attributable to RSV or attributable to influenza was estimated by subtracting the prediction where viral activity was set to zero from the overall prediction. The attributable percent (AP) is defined as the viral-attributable incidence divided by the total predicted incidence. 95% confidence intervals were estimated using a seasonal block bootstrap with 1,000 replicates [28].

### Post-PCV7 Changes in RSV Incidence

We quantified changes in the incidence of RSV, pneumococcal pneumonia, and pneumococcal septicemia hospitalizations following PCV7 introduction in 2000. The change in incidence in each post-vaccine year (2000/2001–2008/2009) in each state was estimated by dividing the yearly incidence by the average pre-vaccine incidence (1997/1998–1999/2000). To obtain an overall estimate for vaccine-associated changes across all states, we fit a negative binomial regression model where the outcome variable was the yearly incidence of RSV, pneumococcal pneumonia, or pneumococcal septicemia, and the predictors were a categorical variable for early or late post-vaccine period (compared to the pre-vaccine reference period) and a categorical variable for state. The incidence rate ratio (IRR) was calculated as the exponent of the regression coefficient for the post-PCV7 period. To get estimates for individual years, we used a categorical variable for the number of years post-PCV7 (rather than a variable for early or late post-PCV7 period).

To estimate how these changes in incidence would translate into cases averted, we multiplied the average number of RSV cases in our study area for 1997/1998–1999/2000 among children aged <2 mo, 3–11 mo, and 12–23 mo by the post-vaccine decline (i.e., 1 − IRR) observed in each age group for 2004/2005–2008/2009. This quantity gives an estimate for the number of cases averted within our study area for each age stratum. This value was then multiplied by the average population in each age stratum for the entire United States (1997–1999) and divided by the average population in our study area to get an estimate of the cases averted per year across the entire US.

In a sensitivity analysis, we evaluated changes since 1992/1993 in the incidence of hospitalizations coded as bronchiolitis, rather than changes in the incidence of hospitalizations with an RSV-specific code (these categories are not mutually exclusive). This analysis was performed because the bronchiolitis codes are less likely than the RSV codes to be biased by changes in viral testing or coding and because reliable bronchiolitis data were available since 1992/1993 (rather than 1997/1998 for RSV).

## Results

### Study Characteristics

The State Inpatient Databases used for these analyses include data from 36 states from 1992/1993 to 2008/2009 (Figure S1). Every hospitalization occurring at a community hospital in the included states is captured in this database, but some states contributed fewer years of data (Figure S1). For 2008/2009, there were 6.7 million children aged <2 y living in the states included in the database. For the period from 1997/1998 to 2008/2009, there were 70.2 million person-years of observation for the < 2-y-old children, including 11,600 hospitalizations coded as pneumococcal pneumonia and 5,804 hospitalizations coded as pneumococcal septicemia (Table 1). Additionally, there were 729,526 hospitalizations coded as RSV (since 1997/1998) among < 2-y-old children (840,464 across all age groups) and 70,872 coded as influenza among < 2-y-old children (434,034 across all age groups). There were 977,417 hospitalizations coded as bronchiolitis among < 1-y-old children (3.0 million bronchiolitis hospitalizations across all age groups).

**Table 1:** Study characteristics.

Dataset	Age	Number of Admissions					Population (Person-Years/1,000,000)
		Pneumococcal Pneumonia	Pneumococcal Septicemia	RSV	Bronchiolitis	Influenza	
**Data from all available states**	0–24 mo	11,600	5,804	729,526	—	70,872	70.2
	0–11 mo	6,078	3,402	610,871	977,417	48,586	35.3
	12–23 mo	5,522	2,402	118,655	—	22,286	34.9
	All ages (incl. adults)	—	—	840,464	—	434,034	2,549.8
**Data from states that could be stratified by age 0–2 and 3–11 mo** †	0–2 mo	828	436	113,716	—	—	3.5
	3–11 mo	1,374	921	113,652	—	—	10.4

Number of admissions for each condition in the included states and cumulative population residing in those states for 1997/1998–2008/2009.

† Arizona, California, Colorado, Georgia, Iowa, Kansas, Massachusetts, New York, and South Carolina. See Table S1 for the tabulation of cases by state.

### Seasonality of RSV, Influenza, and Pneumococcal Pneumonia

**Figure 1:**
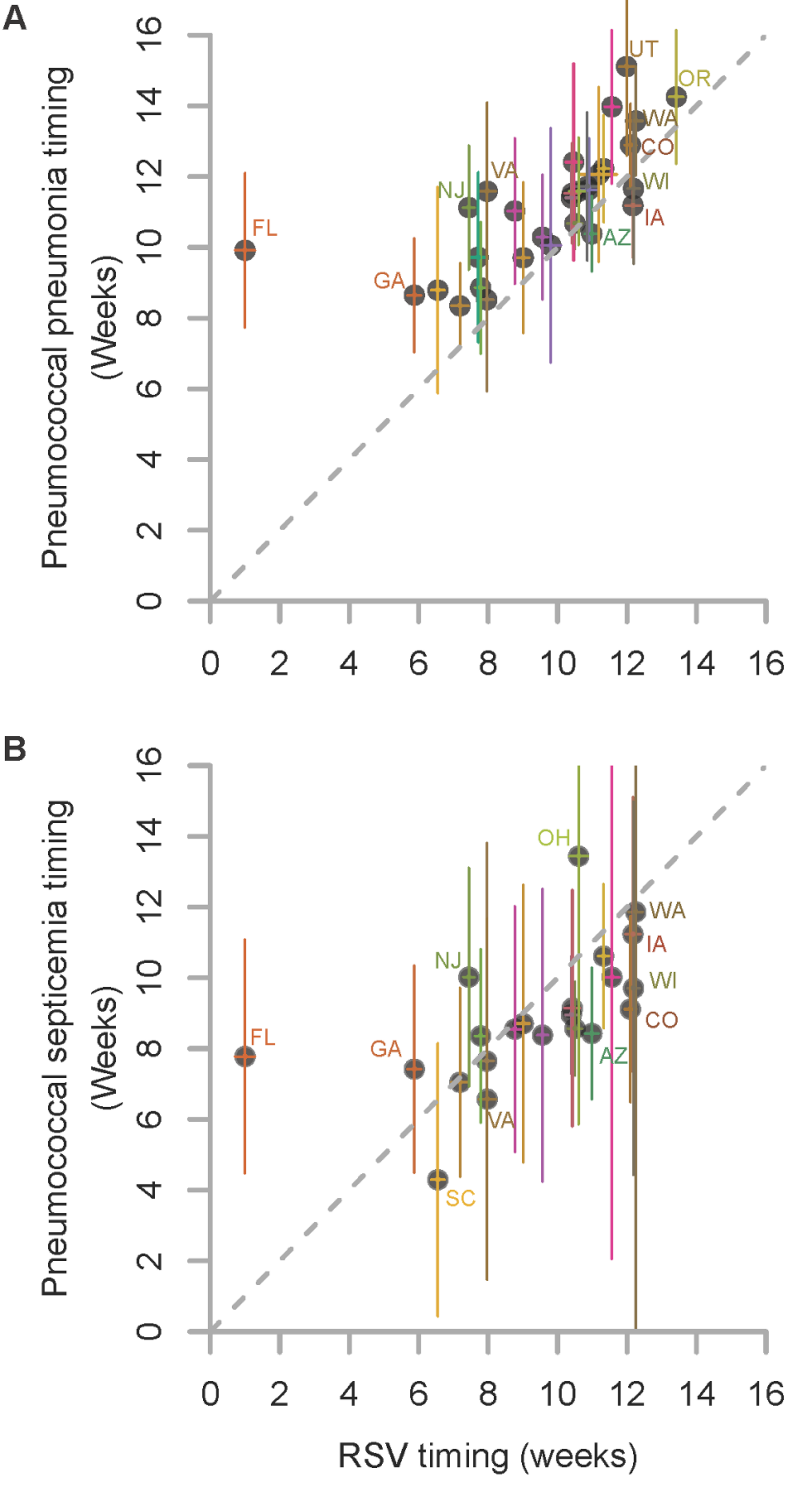
Relationship in the timing of the average seasonal peak of RSV, pneumococcal pneumonia, and pneumococcal septicemia. Association between the average peak timing (in weeks) of RSV hospitalizations and the average peak timing (in weeks) of (A) pneumococcal pneumonia hospitalizations and (B) pneumococcal septicemia hospitalizations in each state among children aged <2 y, 1992/1993–2008/2009. Smaller values indicate earlier epidemics. The error bars indicate the 95% confidence intervals. The colors differentiate the states; labels for selected states are shown.

**Figure 2:**
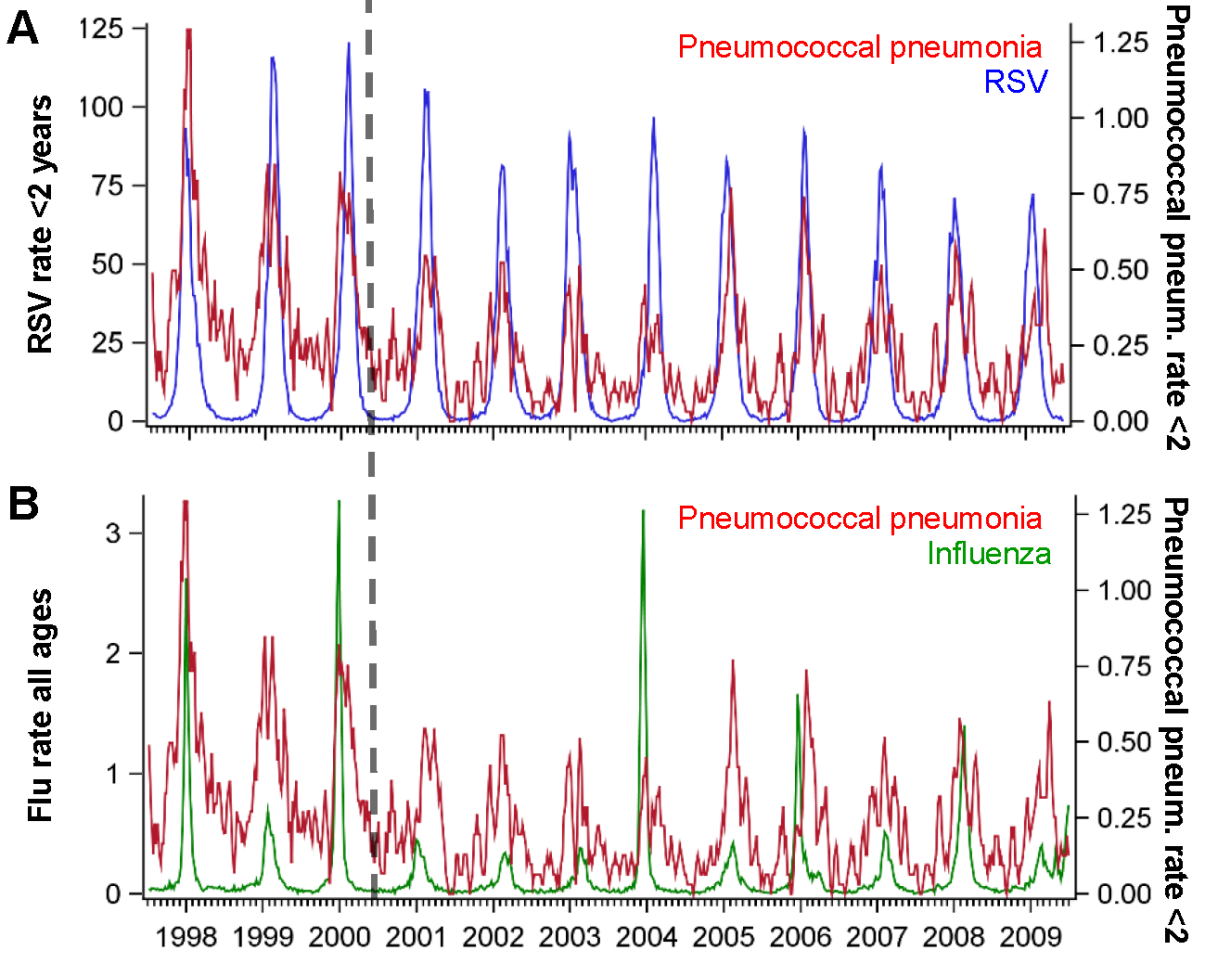
Time series of hospitalizations for pneumococcal pneumonia, RSV, and influenza in California. Incidence rate of pneumococcal pneumonia (red, 3-wk moving average) among children age <2 y in California compared with the incidence of (A) RSV among children aged <2 y (blue) and (B) influenza among all ages (green), 1997/1998–2008/2009. The *x*-axis shows the year and quarter. Incidence is defined as cases per 100,000 children.

The weekly incidences of RSV and pneumococcal pneumonia hospitalizations among < 2-y-old children exhibited strong seasonal variations, with peaks occurring from autumn through the winter (Figures 1 and 2A). RSV exhibits a distinctive spatiotemporal pattern, with earlier epidemics in the southern and eastern United States and later epidemics in the northern and western states (Figure 1). Influenza epidemics in the United States also exhibit winter seasonality, with strong year-to-year variations in intensity (Figure 2B).

### Spatiotemporal Association between RSV Activity and Pneumococcal Hospitalization

We considered whether there was a relationship between the timing of the peaks of seasonal RSV epidemics and the timing of the peaks of pneumococcal pneumonia or septicemia hospitalizations in each state. Pneumococcal pneumonia hospitalizations in < 2-y-old children exhibited a spatiotemporal pattern that was similar to that of RSV hospitalizations. There was a strong correlation between the average peak timing across the US of RSV hospitalizations and pneumococcal pneumonia hospitalizations (ρ = 0.70, 95% CI: 0.45, 0.84; Figure 1). On average, the epidemic timing of pneumococcal pneumonia lagged behind the RSV epidemics by 1.5 wk (95% CI: 0.8, 2.2; Figure 1). The outlier was Florida, where there was a relatively flat, early RSV peak, and pneumococcal pneumonia cases in children peaked substantially later. There was also a significant association between the timing of RSV hospitalizations and the timing of pneumococcal septicemia hospitalizations (ρ = 0.59, 95% CI: 0.21, 0.80; Figure 1), but there was no detectable lag between the RSV and pneumococcal septicemia epidemic peaks. There was notably more uncertainty about the timing of the pneumococcal septicemia epidemics because of sparser data (Figure 1).

### Association between RSV and Influenza Activity and Pneumococcal Pneumonia Incidence

We next considered whether seasonal increases in RSV activity were associated with variations in pneumococcal pneumonia incidence. Using regression models that controlled for shared seasonal factors and influenza activity, we evaluated the association between RSV and pneumococcal pneumonia hospitalizations. Among < 1-y-old infants, 20.3% (95% CI: 17.4%, 25.1%) of pneumococcal pneumonia cases were associated with RSV activity (IRR: 1.23, 95% CI: 1.19, 1.30). Further stratification by age revealed that this association was particularly pronounced in children aged 0–2 mo, where 35.7% (95% CI: 27.9%, 42.7%) of pneumococcal pneumonia cases were associated with RSV (Table 2; Text S1) (IRR: 1.42, 95% CI: 1.30, 1.55). In children aged 3–11 and 12–23 mo, the estimated association with RSV was smaller but still significant (AP: 20.0% [95% CI: 14.7%, 24.8%] and 10.1% [95% CI: 7.6%, 13.9%], respectively; Table 2) (respectively, IRR: 1.24, 95% CI: 1.17, 1.33; IRR: 1.12, 95% CI: 1.09, 1.18). The association between RSV and pneumococcal septicemia hospitalizations was substantially smaller than the association between RSV and pneumococcal pneumonia hospitalizations, with AP estimates of 0.1%–15.0%, with the strongest effect in children aged 0–2 mo (Table 2).

**Table 2:** Percent of pneumococcal pneumonia and pneumococcal septicemia cases attributable to RSV and influenza in children aged <2 y, 1997/1998–2008/2009.

Disease	Age	RSV AP (95% CI)	Influenza AP (95% CI)
**Pneumococcal pneumonia**	**0–11 mo**	20.3 (17.4, 25.1)	0.6 (−0.9, 1.4)
	0–2 mo	35.7 (27.9. 42.7)	2.1 (−4.5, 7.9)
	3–11 mo	20.0 (14.7, 24.8)	2.2 (0.1, 3.4)
	**12–23 mo**	10.1 (7.6, 13.9)	3.2 (1.7, 4.7)
**Pneumococcal septicemia**	**0–11 mo**	7.2 (5.3, 9.0)	−0.6 (−1.4, 0.3)
	0–2 mo	15.0 (13.1, 17.1)	0.7 (−1.1, 2.2)
	3–11 mo	0.1 (−4.9, 5.0)	−2.7 (−3.7, −1.7)
	**12–23 mo**	3.8 (2.5, 5.2)	1.9 (1.1, 2.6)

The estimates of the AP are calculated using the results of the regression model by dividing the number of pneumococcal disease cases predicted if RSV were not present by the number of pneumococcal disease cases predicted based on the observed incidence of RSV.

Influenza activity was also associated with significant increases in pneumococcal pneumonia among children aged 3−11 and 12−23 mo (AP: 2.2% [95% CI: 0.1%, 3.4%] and 3.2% [95% CI: 1.7%, 4.7%], respectively; Table 2). While influenza, compared with RSV, was associated with a smaller fraction of all pneumococcal pneumonia cases, the effect was more pronounced when focusing just on seasons with severe influenza epidemics (e.g., 2003/2004; Figure 2). For instance, during 2003/2004, 6.6% (95% CI: 3.1%, 10.0%) of pneumococcal pneumonia cases were associated with influenza among children aged 12−23 mo, compared with 3.2% (95% CI: 1.7%, 4.7%) across all years.

We also explored the sensitivity of the results to different modeling choices, such as using additive versus multiplicative models, removing the seasonal component from the viral variables prior to fitting the model, using different sets of covariates, and using different lags of the viral variables (Text S1). Regardless of model choice, the same age pattern emerged, with larger RSV-associated changes in pneumococcal pneumonia among children aged 0−2 mo, and smaller changes among those aged 1 y or older. However, the magnitude of the AP estimates differed between models. The results from an additive (identity-linked) model were higher than those from the multiplicative (log-linked) model (Figure S2). The association between RSV and pneumococcal pneumonia and the age patterns were confirmed in sensitivity analyses using models that included seasonally adjusted viral incidence rather than raw viral incidence (Figure S3). Variations in how the baseline was modeled had limited influence on the estimates of the AP (Table S1; Figure S4). Models that included raw viral incidence fit the data better than models that included seasonally adjusted viral incidence (Table S1). Lagging the RSV variable resulted in a poorer fit of the model, while lagging the influenza variable had no impact on model fit (Table S2; Figure S5). There is little collinearity between RSV and influenza (*R* ^2^ = 0.13, 95% CI: 0.12, 0.14) (Figure 2). Finally, hospitalizations coded as syndromic bronchiolitis might be less subject to coding biases than hospitalizations coded as RSV. Substituting a weekly bronchiolitis variable (Figure S6) for the weekly RSV variable in the regression resulted in similar estimates (AP: 24.7% [95% CI: 21.1%, 28.9%] for bronchiolitis compared with 20.3% [95% CI: 17.4%, 25.1%] for RSV for < 1-y-old children).

### Changes in RSV Activity following the Introduction of Pneumococcal Conjugate Vaccine

**Figure 3:**
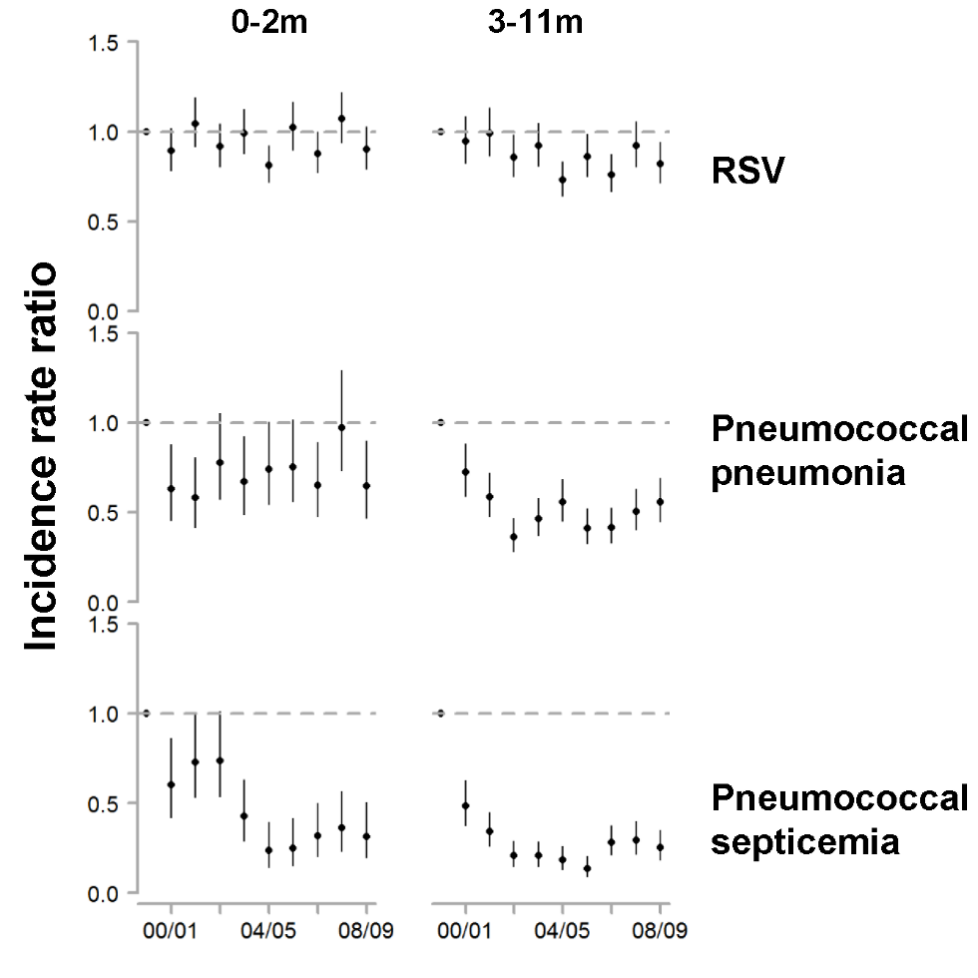
Change in hospitalization rates in each year compared with the average of 1997/1998–1999/2000 among children aged 0–2 and 3–11 mo for RSV, pneumococcal pneumonia, and pneumococcal septicemia. The *y*-axis is the IRR and 95% confidence intervals, with values below one representing declines compared with the baseline period.

**Figure 4:**
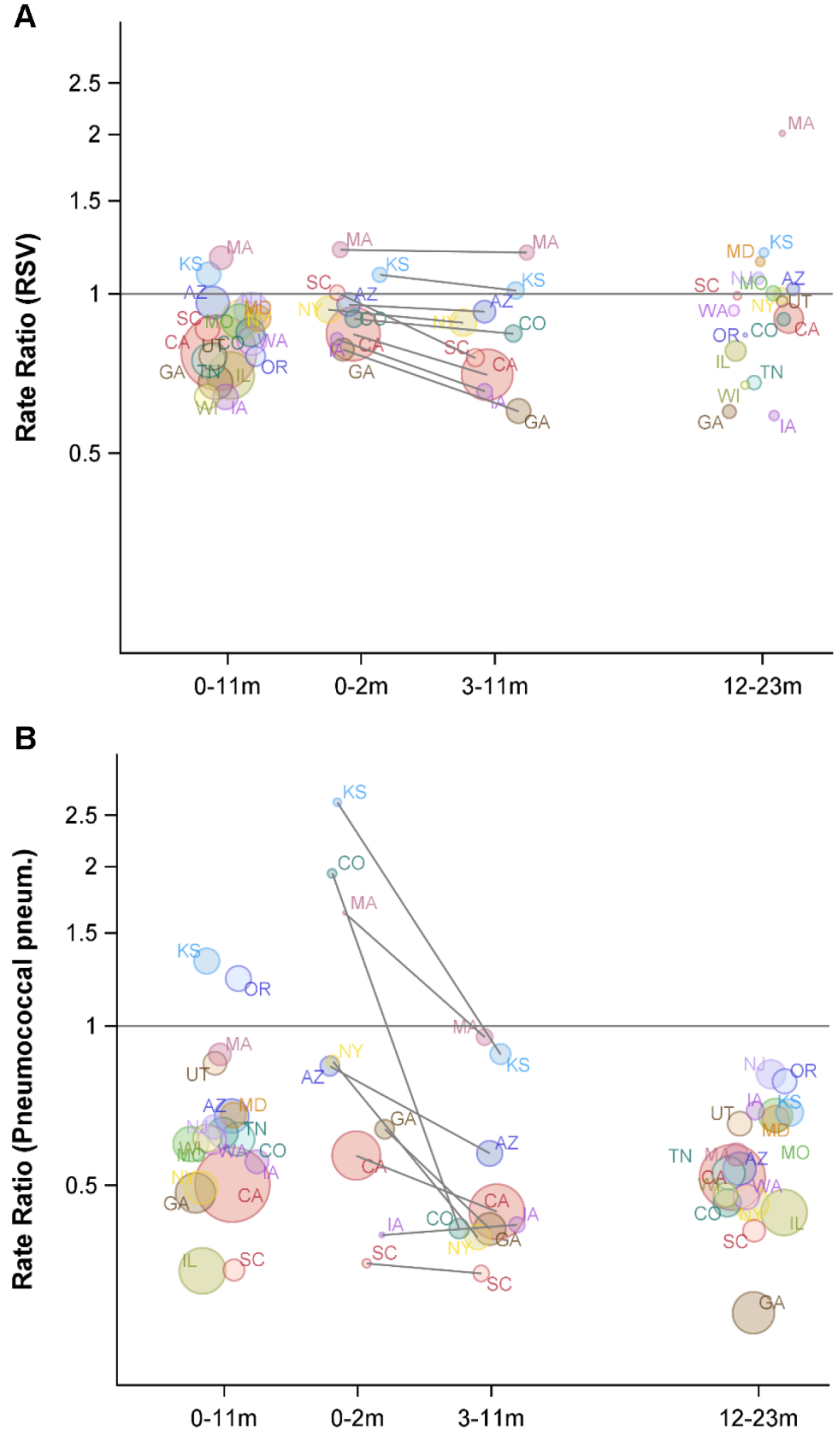
Variations between states and age groups in the change in rates of hospitalization for RSV or pneumococcal pneumonia after introduction of PCV7. Decline in the rate of (A) RSV hospitalizations and (B) pneumococcal pneumonia hospitalizations in each state for 2004/2005−2008/2009 compared with the average of 1997/1998−1999/2000 for children aged 0−11, 0−2, 3−11, and 12−23 mo. The IRRs are shown, with values below one indicating a decline compared with the baseline period. The size of the bubbles is proportional to the inverse variance (i.e., more confidence in larger bubbles). Fewer states were available for the analysis of children aged 0−2 and 3−11 mo. The lines demonstrate the difference in IRRs between children aged 0−2 and 3−11 mo. The colors differentiate the states.

We evaluated whether the incidence of RSV hospitalizations declined in the years following PCV7 introduction. Comparing the incidence of RSV hospitalizations in 2004/2005−2008/2009 with that in the 3 y prior to PCV7 introduction, there was a significant decline among < 1-y-old children (−18.0%, 95% CI: −22.6%, −13.1%) and a smaller decline among children aged 12−23 mo (−9.2%, 95% CI: −15.1%, −2.9%). The decline in RSV was most apparent among children aged 3−11 mo (−18.4%, 95% CI: −25.4%, −10.6%). This decline was evident by 2002/2003 (Figures 3 and 4). In comparison to in children aged 3−11 mo, the decline in RSV incidence in children aged 0−2 mo was more modest and not significant (−6.3%, 95% CI: −14.7%, 2.2%; Figures 3 and 4). These rates of decline among < 2-y-old children would translate to 9,970 (95% CI: 3,232, 15,960) fewer RSV hospitalizations per year if applied to the entire < 2-y-old population in the US (based on the disease rates and population size for 1997−1999).

**Figure 5:**
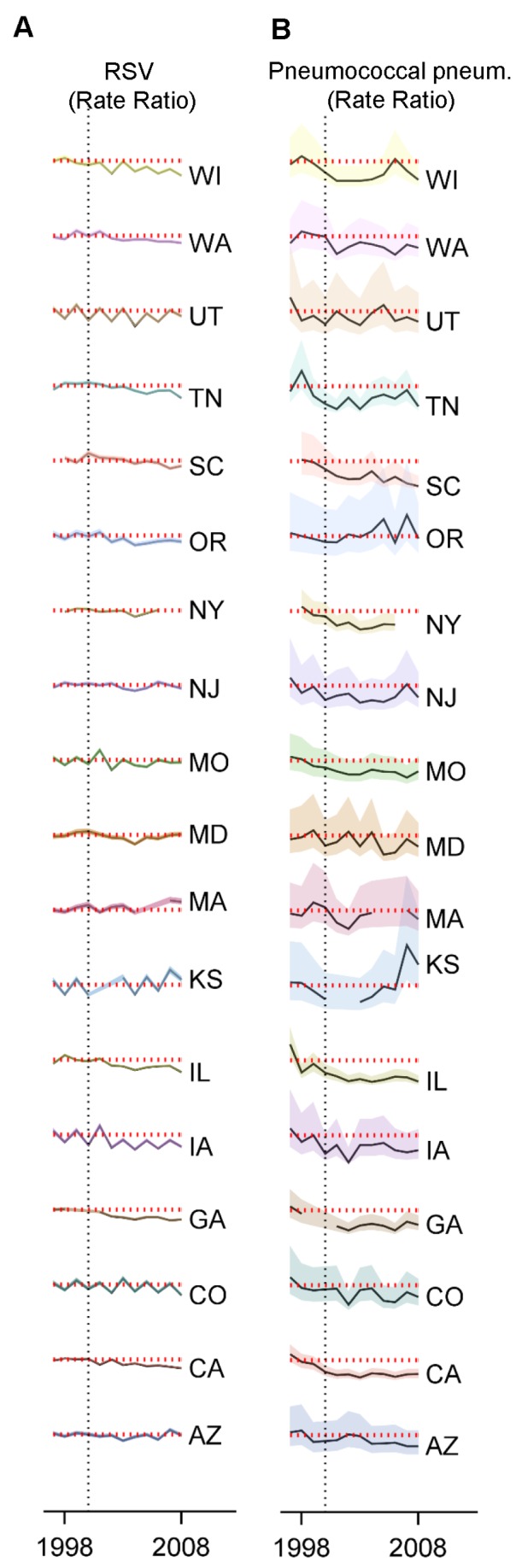
Variations between states, age groups, and years in the change in rates of hospitalization for RSV or pneumococcal pneumonia after introduction of PCV7. Decline in the rate of (A) RSV hospitalizations and (B) pneumococcal pneumonia hospitalizations in each state and each year (July−June) among children aged 0−11 mo compared to the average of 1997/1998−1999/2000 in the same state. The shaded areas indicate the 95% confidence intervals for the IRRs. The red dotted line indicates a rate ratio of one (no change). The colors differentiate the states.

As a comparison, we also evaluated the timing of the declines in pneumococcal septicemia and pneumococcal pneumonia cases after PCV7 introduction. There were sharp and immediate declines in the incidence of pneumococcal septicemia among the children aged 0−2 and 3−11 mo starting in 2000/2001 (Figure 3). However, similar to the pattern observed for RSV, pneumococcal pneumonia incidence declined more among children aged 3−11 mo than among children aged 0−2 mo (Figures 3 and 4). The declines in pneumococcal pneumonia and septicemia incidence were detectable sooner than the decline in RSV incidence. There was considerable variability in the magnitude of the estimated change between states for both RSV and pneumococcal pneumonia (Figures 4 and 5).

Finally, we considered whether the results were sensitive to the choice of the baseline period. RSV coding changed in 1997, preventing comparisons with earlier years. However, there is a strong correlation between weekly hospitalizations coded as syndromic bronchiolitis and those coded as RSV (*R* ^2^ = 0.99, 95% CI: 0.98, 0.99; Figure S6). Between the pre-vaccination era, 1997/1998−1999/2000, and the late post-PCV7 period, 2004/2005−2008/2009, bronchiolitis hospitalizations declined by −15.9% (95% CI: −21.8%, −9.5%) among children aged 3−11 mo. Using an extended pre-vaccine baseline (1992/1993−1999/2000) to estimate post-PCV7 changes in incidence gave a smaller but still significant decline (−12.2%, 95% CI: −18.3%, −5.6%).

## Discussion

Using hospitalization records from a large database covering the majority of states in the United States, we have found evidence supporting an association between pneumococcus and RSV infections. Seasonal epidemics of hospitalizations due to RSV and pneumococcal pneumonia shared a distinctive spatiotemporal pattern, variations in RSV activity were associated with substantial subsequent increases in pneumococcal pneumonia incidence, and RSV activity declined significantly among children aged 3−11 mo after pneumococcal conjugate vaccine (PCV) introduction. These results, obtained from population-based epidemiological data, suggest that RSV might increase the risk for pneumococcal infections and that a subset of hospitalized RSV cases might have a mixed viral–bacterial etiology. These findings are unique in that we were able to use data from an unusually large and robust database to perform detailed age-stratified analyses, to evaluate spatiotemporal patterns across a large area, and to control for seasonality using harmonic regression models. Further studies relying on individual-level data from pneumonia patients will help to clarify the importance of this association.

Our findings are consistent with some previous studies demonstrating a link between RSV and pneumococcus. Children hospitalized with RSV had a higher risk for developing invasive pneumococcal infections [10], and a randomized controlled trial demonstrated that children aged >4 mo who received PCV had lower rates of RSV hospitalizations than those who did not [13]. The magnitude of the observed change in RSV hospitalization rates among children aged 3−11 mo from our study is comparable to that found in the randomized controlled trial of PCV [13]. Likewise, a study from Alaska found that RSV pneumonia rates were declining in recent years, with the authors suggesting that the decline could be due to RSV prophylaxis or possibly to PCV7 [29]. A previous study found a link between bronchiolitis admissions (a proxy for RSV disease) and invasive pneumococcal pneumonia cases in children [11]. Other studies also identified a correlation between the seasonal timing of RSV and pneumococcal disease epidemics [4],[18],, although some of these previous analyses did not control for the shared winter seasonality of the epidemics, which is critical for proper interpretation. A recent study in England and Wales [17] found no association between RSV and invasive pneumococcal disease among children aged <5 y. In our study, the association was weaker for pneumococcal septicemia than for pneumococcal pneumonia and was weaker in older children. Therefore, we suggest that future analyses in this area should account for the clinical syndrome of the invasive pneumococcal disease cases and should focus on younger children.

Mechanistically, these results support the notion that a primary RSV infection increases the risk for a secondary pneumococcal pneumonia infection. Recent experimental work demonstrates that RSV can directly bind to pneumococcal surface proteins and influence the virulence of the bacteria [15]. With such a direct mechanism, we would expect that RSV activity would increase first, and pneumococcal disease rates would increase either at the same time or within a short time period. The ∼1.5-wk lag between the epidemic peaks of RSV and pneumococcal pneumonia is consistent with such a mechanism.

The observed decline in RSV hospitalizations could be consistent with the notion that some fraction of the cases diagnosed with RSV have a mixed bacterial–viral etiology. For example, in some cases, a child could have a mild or asymptomatic RSV infection, pneumococcus makes it severe and leads to hospitalization, but only RSV infection is diagnosed. In such a scenario, elimination of virulent pneumococci with PCVs would reduce the rate of diagnosed RSV hospitalizations.

The finding of a relationship between RSV and pneumococcal disease seemingly contradicts some previous studies that suggested that secondary bacterial infections do not commonly follow RSV [16],[30]. However, this issue can be resolved by considering that there are actually two separate but related questions. The first question is whether RSV and pneumococcus interact, and whether this synergism leads to higher rates of hospitalization attributed to one or both pathogens. This issue is important for determining what interventions might prevent hospitalizations caused by RSV or respiratory bacteria. Our results support the notion that these pathogens do interact and that some “RSV hospitalizations” might have a mixed viral–bacterial etiology.

The second question is whether children hospitalized with RSV are at increased risk for developing a secondary bacterial infection that could be prevented or treated with antibiotics. Studies of this issue have reported seemingly conflicting findings. Some have demonstrated that RSV cases do not have subsequent bacteremia [16], but others have found evidence of a relationship between RSV and subsequent bacterial infections [10],[31]. This apparent conflict could be a matter of these studies considering the absolute versus relative risk. There are many RSV hospitalizations in children, but comparatively few cases of bacteremia (and bacteremia is an insensitive method for detecting bacterial infections). It is possible that a large percentage of bacterial pneumonia cases could be attributable to RSV (as our data suggest for young children), but a smaller percentage of RSV cases might have bacterial co-infections. Additionally, the inflammatory damage caused by the secondary bacterial infection could occur prior to a child visiting a clinician. Such a phenomenon could explain why clinical trials of antibiotics to treat bronchiolitis have shown limited effects [32]. Our data suggest that bacterial co-infection could be considered in children hospitalized with pneumonia (rather than bronchiolitis), even those who test positive for RSV infection, and antibiotic treatment might be appropriate in some instances.

The age pattern observed here—with a moderate post-PCV7 decline in RSV and pneumococcal pneumonia in children aged 3−11 mo but no change in RSV among children aged <3 mo—is somewhat surprising but is consistent with one prior study that showed that PCV7 had limited impact in children aged <3 mo because of higher levels of serotype replacement [25]. Likewise, our finding of a significant decline in pneumococcal septicemia is consistent with studies that detected a decline in invasive pneumococcal disease among neonates [33],[34]. There are several potential explanations for these patterns. First, it is possible that pneumococcal septicemia and pneumococcal pneumonia have different risk factors: the children who get sick with pneumococcal pneumonia might be susceptible to whatever pneumococcus they are exposed to, regardless of serotype, so increases in carriage of non-vaccine serotypes might have a large impact. In contrast, for the children who get sick with pneumococcal septicemia, the specific serotype they are exposed to might be more important, so exposure to the less invasive non-vaccine serotypes might not increase the risk for disease. Additionally, administration of nine-valent PCV in children at 6 and 14 wk of age has been temporally associated with an increased risk of RSV hospitalization [35].

The data used in our analyses end in 2009, just prior to the introduction of PCV13 in the United States. This next-generation pneumococcal vaccine has further decreased the incidence of pneumococcal disease [36]. However, we expect that the interaction between pneumococcus and RSV will still be important. The vaccine has likely changed the distribution of serotypes causing disease in children. However, pneumococcus is still commonly carried among healthy children [37], and recent evidence suggests that RSV directly binds to a conserved pneumococcal surface protein present in all serotypes [15]. Therefore, we expect that RSV will still be associated with increases in the incidence of pneumococcal disease caused by non-vaccine serotypes, even if the overall incidence of pneumococcal disease has declined.

It will be necessary to repeat these types of analyses in resource-poor countries to understand the relative importance of co-infections in these settings. The burdens of both RSV and pneumococcal disease are particularly high in resource-poor settings [9],[38]. Bacterial–viral interactions could be particularly important in understanding and quantifying the impacts of vaccines against pneumococcus, which are now being deployed in many resource-poor settings around the world. For instance, studies that focus on pathogen-specific outcomes (e.g., pneumococcal septicemia) will likely underestimate the full impacts of the vaccine. Likewise, future interventions targeting RSV (e.g., maternal immunization) could have secondary effects against pneumococcus or other bacterial pathogens. Understanding the strength of these interactions could help to predict the full impact of an RSV vaccine program.

The strength of the association between RSV and pneumococcus could differ in resource-poor settings. It is possible that host factors, such as immunodeficiencies or nutritional problems, contribute more to the burden of pneumococcal disease in developing countries, making the relative contribution of respiratory viruses less or more important (e.g., a smaller or larger AP). Further time series studies in resource-poor settings with high-quality data, such as in South Africa [13],[39],[40], could help to clarify this interaction in different risk groups.

Other studies on this topic have used weather variables to control for shared seasonal timing [12],[17],[41] rather than harmonic variables, which was the approach used here. Since there is not a clear causal link between weather and RSV or pneumococcal disease (and if there is a link, it could be nonlinear), we used harmonic variables to avoid introducing an additional source of uncertainty. However, there could be other confounders not captured by the harmonic terms that could influence the results. For instance, dynamic processes (such as waning immunity) could be synchronized by a common environmental risk factor and could lead to similar epidemic timing for RSV and pneumococcus.

Our study and interpretations have several caveats. These analyses define disease rates on the basis of hospitalization discharge codes rather than direct microbiological evidence. As a result, it is possible that cases coded as pneumococcal pneumonia/lobar pneumonia might not have a pneumococcal etiology. If some of the pneumococcal pneumonia cases are, in fact, RSV cases without a bacterial co-infection, then this would lead to higher estimates of AP. Few RSV cases result in lobar pneumonia [42] (which shares an ICD-9 code with pneumococcal pneumonia), decreasing the chances of such misclassification. While ICD-9 codes for pneumococcal pneumonia and septicemia are sensitive and specific in some settings [43], they have not been specifically validated in children or following vaccine introduction. Therefore, potential misclassification of cases presents an important source of uncertainty that we are not able to capture with our models. The estimates of post-vaccine changes in disease incidence are based on population-level data and could be subject to confounding by changes in demographics, medical practice, viral testing, and discharge coding. Additionally, changes in the use of palivizumab in high-risk children could also have reduced RSV hospitalization rates over time. Therefore, our results will need to be replicated with different study designs (such as a prospective cohort study) to determine whether there is a causal link between PCV introduction and RSV incidence.

The proxies that we use for viral activity—hospitalizations coded as RSV or influenza—are subject to limitations. The number of hospitalizations coded for influenza or RSV will almost certainly underestimate the true number of cases [41]. However, for the purposes of regression modeling, the timing and amplitude of the epidemics—rather than their absolute magnitude—would influence the results. We would not expect biases in coding to influence the relative timing or amplitude of the epidemics. Because of known biases in influenza testing and coding by age group, we used influenza cases aggregated across all age groups. We would expect that epidemic timing would not vary substantially between age groups in a given location [44], so this is unlikely to influence our results.

Finally, all of these analyses rely on data aggregated into broad categories. Such ecological analyses are subject to potential biases and should be interpreted with caution. While the results support the hypothesis that RSV and pneumococcus interact, further individual-level studies of pediatric pneumonia cases would help to support these findings. Given our results here, we would expect to find the greatest evidence of an interaction between RSV and pneumococcus among children aged <1 y, and particularly among neonatal pneumonia cases. One approach would be to look for evidence of a recent RSV infection among neonatal pneumonia cases with confirmed evidence of pneumococcal disease (or other bacterial pneumonias) and compare these cases to an appropriate control group.

In conclusion, our analyses suggest that there is a relationship between RSV and pneumococcal pneumonia hospitalizations, supporting previous observations in a randomized clinical trial [13]. RSV is associated with increases in the incidence of pneumococcal pneumonia, particularly in young infants, and a percentage of RSV hospitalizations might be attributable to pneumococcus, based on post-PCV7 declines. These findings could help in the design of more effective interventions to treat respiratory infections in young children.

## Supporting Information

Figure S1 **Years and states included in each analysis.** A black mark indicates that the state and year was included in the analysis. The red vertical line indicates the time of PCV7 introduction in the United States. The states are sorted based on the first year available for each analysis. For the pre/post-PCV7 incidence graph, the states highlighted in red had data available for <1-y-old children that could be further stratified by 0–2 and 3–11 mo of age.
(PDF)

Figure S2 **Estimates of the RSV attributable percent in each age group calculated with a multiplicative Poisson model or an additive linear model.** The diagonal line denotes *x* = *y*.
(TIF)

Figure S3 **Estimates of the RSV attributable percent in each age group from a multiplicative model where the RSV variable is raw RSV counts compared with incidence rate ratio estimates from a model that used seasonally adjusted RSV counts.** Raw RSV counts (*y*-axis); seasonally adjusted RSV counts (*x*-axis).
(TIF)

Figure S4 **Estimates of the RSV attributable percent from different candidate models.** The size of the circle is proportional to the BIC weights, and the number indicates which model was used to generate the estimate (see Table S1 for model numbers).
(TIF)

Figure S5 **Estimates of the RSV and influenza attributable percent in each age group from a multiplicative model where the influenza and RSV variables were lagged as in** **Table S2** **.** The similarity of the estimates indicates that all of the well-fitting models produced similar estimates of the AP. The numbers on the graph indicate the model used (from Table S2) to calculate the AP.
(TIF)

Figure S6 **Incidence of RSV compared with the incidence of bronchiolitis among children aged <1 y in Iowa, 1997/1998–2008/2009.** RSV (blue); bronchiolitis (red). The *x*-axis shows the year and quarter. Incidence is defined as cases per 100,000 children.
(PDF)

Table S1 **Comparison of the candidate models that were tested.** “X” indicates that the covariate was included. The BIC scores show the BIC in each age group. Models 1–18 include unadjusted RSV and influenza incidence, while models 19–36 include the seasonally adjusted incidence. The models with the lowest BIC score for each age group are highlighted.
(DOCX)

Table S2 **Effect of lagging the RSV and influenza variables on the fit of the models.** Lower BIC scores indicate better fit. A lag of zero indicates that the viral time series and the pneumococcal pneumonia time series are synchronous. A negative value indicates that the pneumococcal pneumonia time series follows the viral time series by *n* weeks. Models were fit to pneumococcal pneumonia data from children aged 0–11 mo.
(DOCX)

Text S1 **Supplementary analyses include sensitivity analyses related to the choice of the model structure and lags of the viral incidence variables.**
(DOCX)

## Abbreviations

AP: attributable percent
BIC: Bayesian information criterion
ICD-9: International Classification of Diseases, Ninth Revision
IRR: incidence rate ratio
PCV: pneumococcal conjugate vaccine
PCV13: 13-valent pneumococcal conjugate vaccine
PCV7: seven-valent pneumococcal conjugate vaccine
RSV: respiratory syncytial virus
