# Supplementary figures and images for: Association between Respiratory Syncytial Virus Activity and Pneumococcal Disease in Infants: A Time Series Analysis of US Hospitalization Data
Source: PLoS Med. 2015 Jan 6;12(1):e1001776. doi: 10.1371/journal.pmed.1001776 (PMC4285401; doi:10.1371/journal.pmed.1001776)

FIGURE S1.

Attributable  
percent  
estimates

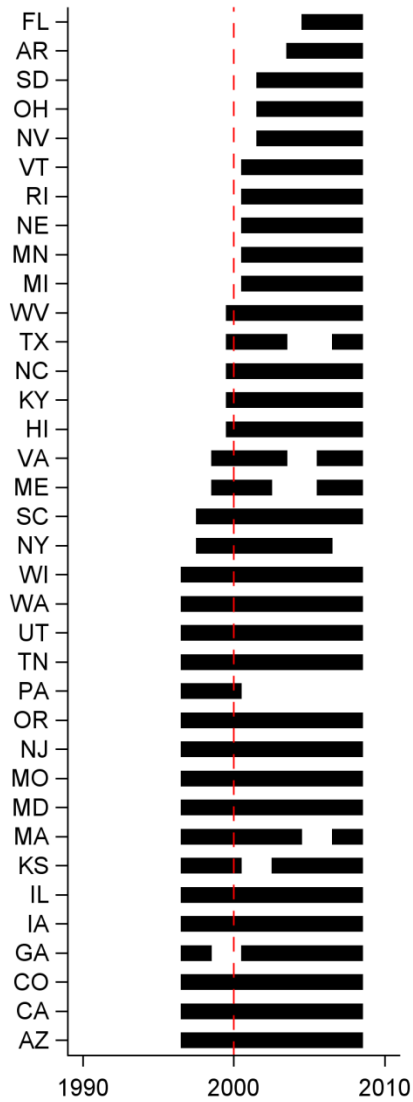

Timing estimates

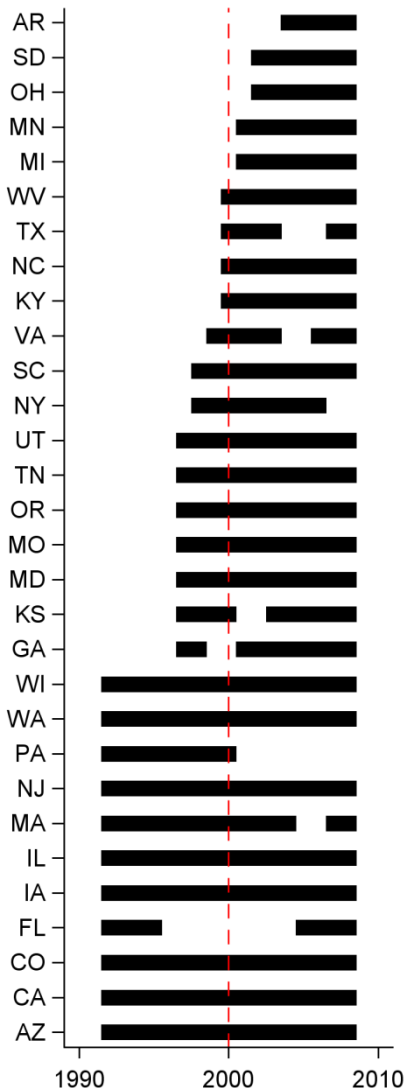

Pre/post-PCV7  
incidence

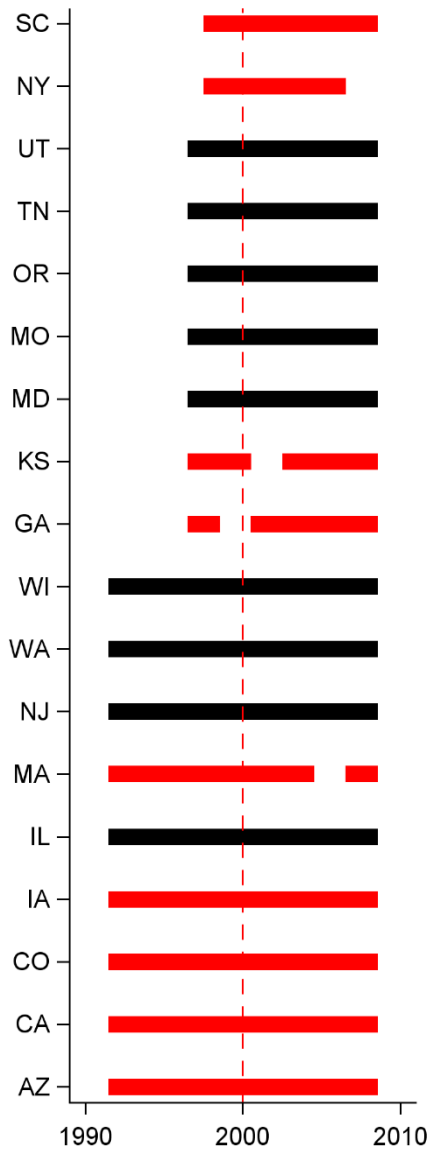

Supplement: Figure S1 — Years and states included in each analysis. A black mark indicates that the state and year was included in the analysis. The red vertical line indicates the time of PCV7 introduction in the United States. The states are sorted based on the first year available for each analysis. For the pre/post-PCV7 incidence graph, the states highlighted in red had data available for <1-y-old children that could be further stratified by 0–2 and 3–11 mo of age. (PDF) [file pmed.1001776.s001.pdf]

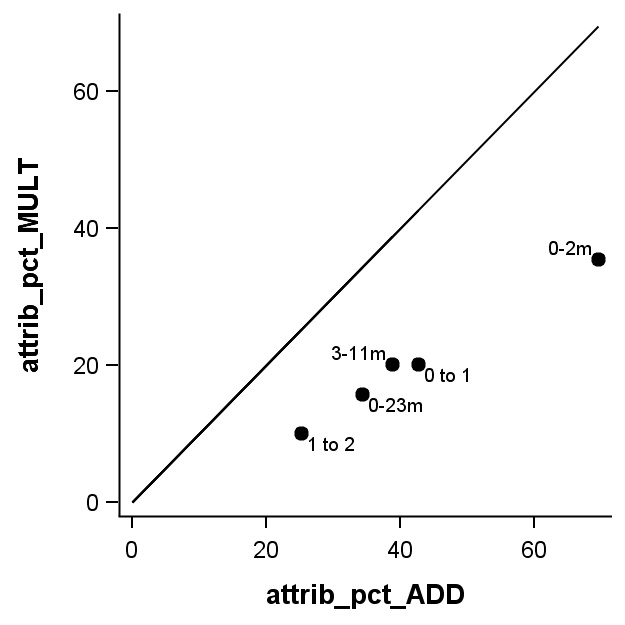

Supplement: Figure S2 — Estimates of the RSV attributable percent in each age group calculated with a multiplicative Poisson model or an additive linear model. The diagonal line denotes x = y. (TIF) [file pmed.1001776.s002.tif]

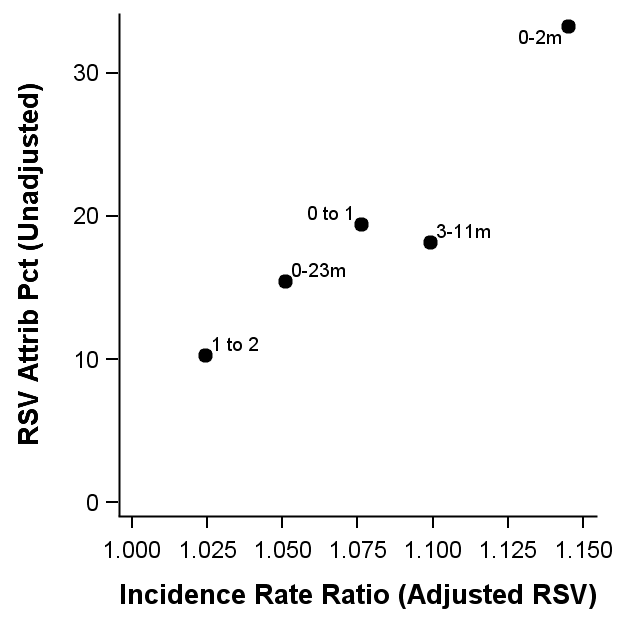

Supplement: Figure S3 — Estimates of the RSV attributable percent in each age group from a multiplicative model where the RSV variable is raw RSV counts compared with incidence rate ratio estimates from a model that used seasonally adjusted RSV counts. Raw RSV counts (y-axis); seasonally adjusted RSV counts (x-axis). (TIF) [file pmed.1001776.s003.tif]

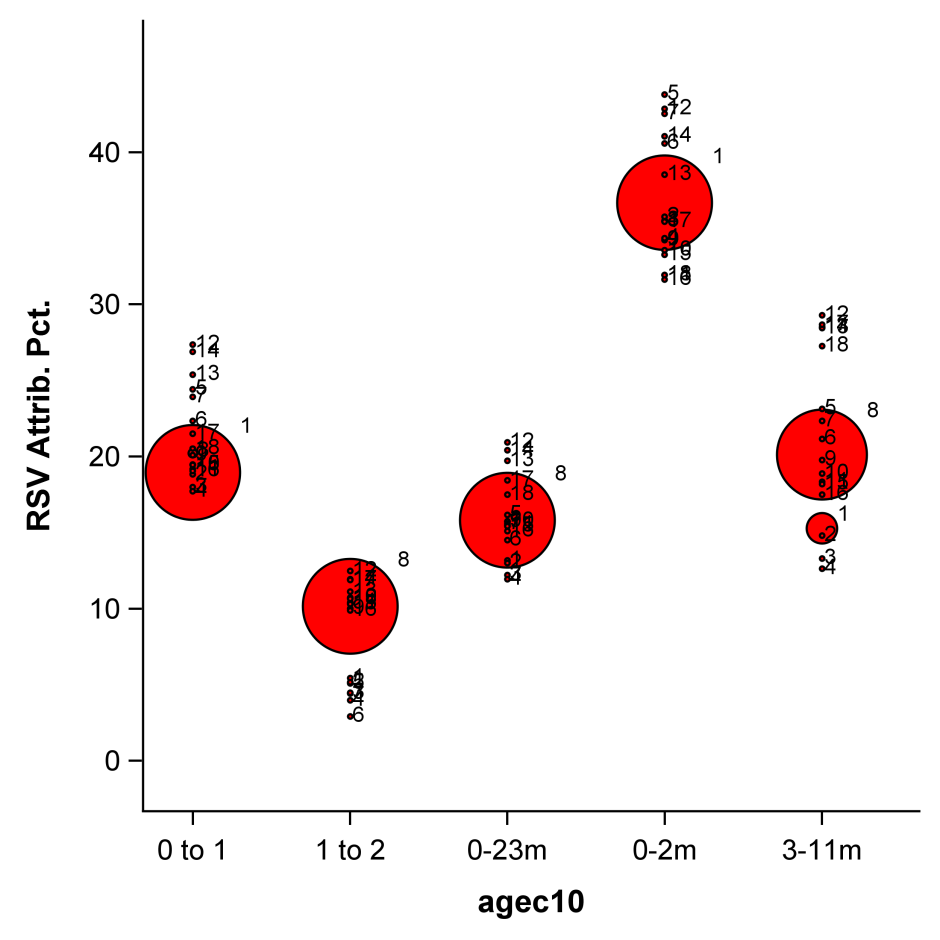

Supplement: Figure S4 — Estimates of the RSV attributable percent from different candidate models. The size of the circle is proportional to the BIC weights, and the number indicates which model was used to generate the estimate (see Table S1 for model numbers). (TIF) [file pmed.1001776.s004.tif]

FIGURE S6.

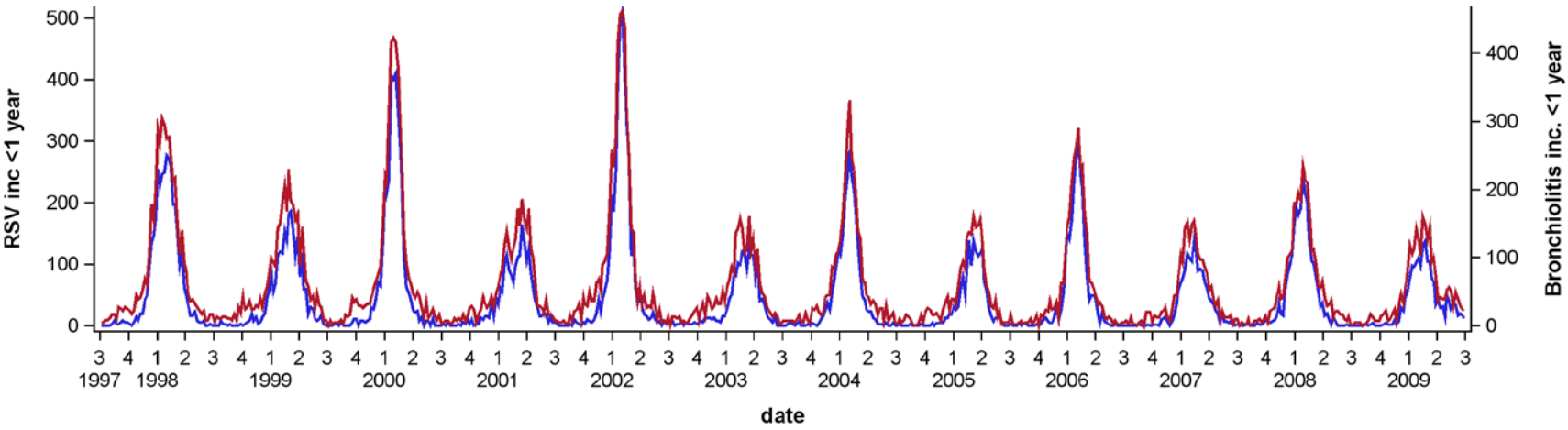

Supplement: Figure S6 — Incidence of RSV compared with the incidence of bronchiolitis among children aged <1 y in Iowa, 1997/1998–2008/2009. RSV (blue); bronchiolitis (red). The x-axis shows the year and quarter. Incidence is defined as cases per 100,000 children. (PDF) [file pmed.1001776.s006.pdf]
