# Supplementary material for: Association between Respiratory Syncytial Virus Activity and Pneumococcal Disease in Infants: A Time Series Analysis of US Hospitalization Data
Source: PLoS Med. 2015 Jan 6;12(1):e1001776. doi: 10.1371/journal.pmed.1001776 (PMC4285401; doi:10.1371/journal.pmed.1001776)
Supplement: Table S1 — Comparison of the candidate models that were tested. “X” indicates that the covariate was included. The BIC scores show the BIC in each age group. Models 1–18 include unadjusted RSV and influenza incidence, while models 19–36 include the seasonally adjusted incidence. The models with the lowest BIC score for each age group are highlighted. (DOCX) [file pmed.1001776.s007.docx]

| Table S1. Candidate models. “X” indicates that the covariate was included. The BIC scores show the BIC in each age group. Models 1-18 include unadjusted RSV and influenza incidence while models 19-36 include the seasonally-adjusted incidence. The model with the lowest BIC score for each age group are highlighted | | | | | | | | | | | | | | | |
| --- | --- | --- | --- | --- | --- | --- | --- | --- | --- | --- | --- | --- | --- | --- | --- |
|  |  |  |  |  |  |  |  |  |  |  | ***BIC SCORES*** | | | | |
| ***Model*** | ***12mo harmonic*** | ***6mo harmonic*** | ***Flu*** | ***RSV*** | ***Vaccine period*** | ***State*** | ***State*12 mo*** | ***State*6mo*** | ***RSV****  ***state*** | ***Vax Period*state*** | ***0-1y*** | ***1-2y*** | ***0-23m*** | ***0-2m*** | ***3-11m*** |
| ***1*** | **X** |  | **X** | **X** | **X** | **X** |  |  |  |  | 21783.04 | 21281.66 | 31515.81 | 3869.173 | 5722.446 |
| ***2*** | **X** |  | **X** | **X** | **X** | **X** |  |  |  | **X** | 22181.25 | 21692.68 | 31872.28 | 3971.571 | 5838.482 |
| ***3*** | **X** |  | **X** | **X** | **X** | **X** | **X** |  |  |  | 22319.2 | 21832.68 | 32051.15 | 3984.615 | 5847.754 |
| ***4*** | **X** |  | **X** | **X** | **X** | **X** | **X** |  |  | **X** | 22716.79 | 22243.61 | 32406.89 | 4086.226 | 5963.523 |
| ***5*** | **X** |  | **X** | **X** | **X** | **X** |  |  | **X** |  | 22032.56 | 21557.05 | 31758.56 | 3926.158 | 5789.27 |
| ***6*** | **X** |  | **X** | **X** | **X** | **X** | **X** |  | **X** |  | 22596.2 | 22130.88 | 32342.95 | 4041.615 | 5910.725 |
| ***7*** | **X** |  | **X** | **X** | **X** | **X** |  |  | **X** | **X** | 22432.11 | 21974 | 32120.99 | 4026.701 | 5905.287 |
| ***8*** | **X** | **X** | **X** | **X** | **X** | **X** |  |  |  |  | 21793.31 | 21231.83 | 31471.75 | 3884.242 | 5718.09 |
| ***9*** | **X** | **X** | **X** | **X** | **X** | **X** |  |  |  | **X** | 22191.98 | 21644.02 | 31829.7 | 3985.914 | 5835.147 |
| ***10*** | **X** | **X** | **X** | **X** | **X** | **X** | **X** |  |  |  | 22331.95 | 21784.44 | 32012.22 | 3999.124 | 5846.476 |
| ***11*** | **X** | **X** | **X** | **X** | **X** | **X** | **X** |  |  | **X** | 22730.23 | 22197.33 | 32370.34 | 4099.777 | 5963.459 |
| ***12*** | **X** | **X** | **X** | **X** | **X** | **X** |  |  | **X** |  | 22029.07 | 21504.61 | 31690.28 | 3942.298 | 5784.432 |
| ***13*** | **X** | **X** | **X** | **X** | **X** | **X** | **X** |  | **X** |  | 22600.14 | 22086.81 | 32289.88 | 4057.317 | 5909.456 |
| ***14*** | **X** | **X** | **X** | **X** | **X** | **X** | **X** | **X** |  | **X** | 22430.3 | 21924.11 | 32057.26 | 4042.161 | 5901.845 |
| ***15*** | **X** | **X** | **X** | **X** | **X** | **X** | **X** | **X** |  |  | 22928.44 | 22388.63 | 32614.94 | 4115.972 | 5968.896 |
| ***16*** | **X** | **X** | **X** | **X** | **X** | **X** | **X** | **X** |  | **X** | 23326.47 | 22801.33 | 32972.62 | 4215.605 | 6085.648 |
| ***17*** | **X** | **X** | **X** | **X** | **X** | **X** | **X** | **X** | **X** |  | 23222.57 | 22688.37 | 32898.41 | 4177.467 | 6032.26 |
| ***18*** | **X** | **X** | **X** | **X** | **X** | **X** | **X** | **X** | **X** | **X** | 23624.26 | 23108.12 | 33268.6 | 4277.108 | 6149.558 |
| ***19*** | **X** |  | **X** | **X** | **X** | **X** |  |  |  |  | 21960.8 | 21293.24 | 31678.84 | 3972.696 | 5743.124 |
| ***20*** | **X** |  | **X** | **X** | **X** | **X** |  |  |  | **X** | 22347.3 | 21703.02 | 32023.73 | 4063.736 | 5855.548 |
| ***21*** | **X** |  | **X** | **X** | **X** | **X** | **X** |  |  |  | 22411.67 | 21830.33 | 32128.93 | 4040.832 | 5850.635 |
| ***22*** | **X** |  | **X** | **X** | **X** | **X** | **X** |  |  | **X** | 22792.76 | 22240.5 | 32472.85 | 4131.713 | 5963.422 |
| ***23*** | **X** |  | **X** | **X** | **X** | **X** |  |  | **X** |  | 22261.38 | 21598.08 | 31978.23 | 4019.065 | 5802.222 |
| ***24*** | **X** |  | **X** | **X** | **X** | **X** | **X** |  | **X** |  | 22704.95 | 22133.6 | 32421.35 | 4094.985 | 5907.549 |
| ***25*** | **X** |  | **X** | **X** | **X** | **X** |  |  | **X** | **X** | 22647.16 | 22008.11 | 32325.53 | 4109.23 | 5914.379 |
| ***26*** | **X** | **X** | **X** | **X** | **X** | **X** |  |  |  |  | 21972.64 | 21276.1 | 31692.93 | 3960.94 | 5754.824 |
| ***27*** | **X** | **X** | **X** | **X** | **X** | **X** |  |  |  | **X** | 22359.55 | 21686.05 | 32037.78 | 4052.351 | 5867.2 |
| ***28*** | **X** | **X** | **X** | **X** | **X** | **X** | **X** |  |  |  | 22423.72 | 21811.35 | 32142.76 | 4036.229 | 5860.873 |
| ***29*** | **X** | **X** | **X** | **X** | **X** | **X** | **X** |  |  | **X** | 22805.51 | 22221.78 | 32486.64 | 4127.415 | 5973.693 |
| ***30*** | **X** | **X** | **X** | **X** | **X** | **X** |  |  | **X** |  | 22274.09 | 21580.3 | 31991.32 | 4012.063 | 5813.871 |
| ***31*** | **X** | **X** | **X** | **X** | **X** | **X** | **X** |  | **X** |  | 22718.35 | 22114.42 | 32434.37 | 4091.619 | 5918.654 |
| ***32*** | **X** | **X** | **X** | **X** | **X** | **X** | **X** | **X** |  | **X** | 22659.82 | 21990.18 | 32338.8 | 4101.489 | 5926.131 |
| ***33*** | **X** | **X** | **X** | **X** | **X** | **X** | **X** | **X** |  |  | 23007.26 | 22407.84 | 32723.53 | 4150.419 | 5980.384 |
| ***34*** | **X** | **X** | **X** | **X** | **X** | **X** | **X** | **X** |  | **X** | 23390.89 | 22818.09 | 33067.53 | 4241.967 | 6093.056 |
| ***35*** | **X** | **X** | **X** | **X** | **X** | **X** | **X** | **X** | **X** |  | 23306.85 | 22709.58 | 33021.93 | 4207.475 | 6039.318 |
| ***36*** | **X** | **X** | **X** | **X** | **X** | **X** | **X** | **X** | **X** | **X** | 23691.08 | 23117.77 | 33367.17 | 4297.287 | 6151.021 |
