# Supplementary material for: Association between Respiratory Syncytial Virus Activity and Pneumococcal Disease in Infants: A Time Series Analysis of US Hospitalization Data
Source: PLoS Med. 2015 Jan 6;12(1):e1001776. doi: 10.1371/journal.pmed.1001776 (PMC4285401; doi:10.1371/journal.pmed.1001776)
Supplement: Table S2 — Effect of lagging the RSV and influenza variables on the fit of the models. Lower BIC scores indicate better fit. A lag of zero indicates that the viral time series and the pneumococcal pneumonia time series are synchronous. A negative value indicates that the pneumococcal pneumonia time series follows the viral time series by n weeks. Models were fit to pneumococcal pneumonia data from children aged 0–11 mo. (DOCX) [file pmed.1001776.s008.docx]

| **Table S2**. Effect of lagging the RSV and influenza variables on the fit of the models. Lower BIC scores indicate better fit. A lag of 0 indicates that the viral time series and the pneumococcal pneumonia time series are synchronous. A negative value indicates that the pneumococcal pneumonia time series follows the viral time series by N-weeks. Models were fit to pneumococcal pneumonia data from 0-11 month old children. | | | |
| --- | --- | --- | --- |
| Model number | RSV lags (weeks) | Flu lags (weeks) | BIC score |
| 1 | 0 | 0 | 20397.8896 |
| 2 | +1 | 0 | 20448.8810 |
| 3 | -1 | 0 | 20417.9609 |
| 4 | -2 | 0 | 20468.8349 |
| 5 | -3 | 0 | 20508.5179 |
| 6 | -4 | 0 | 20547.5396 |
| 7 | 0 | +1 | 20398.0823 |
| 8 | 0 | -1 | 20398.0644 |
| 9 | 0 | -2 | 20398.6651 |
| 10 | 0 | -3 | 20398.7170 |
| 11 | 0 | -4 | 20397.9755 |
|  | | | |
