# Supplementary material for: Association between Respiratory Syncytial Virus Activity and Pneumococcal Disease in Infants: A Time Series Analysis of US Hospitalization Data
Source: PLoS Med. 2015 Jan 6;12(1):e1001776. doi: 10.1371/journal.pmed.1001776 (PMC4285401; doi:10.1371/journal.pmed.1001776)
Supplement: Text S1 — Supplementary analyses include sensitivity analyses related to the choice of the model structure and lags of the viral incidence variables. (DOCX) [file pmed.1001776.s009.docx]

**TEXT S1: Sensitivity analyses**

***Sensitivity of results to model link.***

We considered whether using an additive (identity) link rather than a multiplicative (log) link in the regression models would influence our estimates of the RSV-attributable percent. We fit a linear regression where the outcome variable was weekly incidence of pneumococcal pneumonia, and the predictors were the same as those used in the main log-Poisson model. The relative importance of RSV between age groups was the same in both models (**Figure S2).** However, the attributable percent estimates were consistently higher for the additive model compared with the multiplicative model.

We believe the multiplicative model is preferable for several reasons. First, it readily allows the effect of RSV to scale with the baseline incidence—as pneumococcal disease declines, RSV continues to be an important cause of disease in relative terms, but the absolute rates of pneumococcal disease decline substantially. Such an effect could happen if the pneumococcal serotypes causing disease following vaccination are less invasive (Yildirim 2010). Additionally, because of the convergence issues with the additive model in some strata, we chose to use the log-linked model for the main analysis.

***Adjustment for seasonality in viral predictors***

The analysis above suggests that the estimates of the association between RSV and pneumococcal disease were somewhat sensitive to the model link (additive vs multiplicative). One potential explanation for this is that the RSV term could be “competing” with the sine and cosine terms included in the regression model to explain the seasonal variations. To avoid this problem we attempted to remove the seasonal components from the RSV and influenza variables prior to fitting the model. To do this, we fit Poisson regression models (log-linked), where the outcome variable was either RSV or influenza incidence, and the predictors were sine and cosine terms with 12-month harmonics and a dummy variable for PCV-vaccination period. These models were fit separately for each state. We then divided the observed weekly viral incidence by the expected incidence calculated from each model. This gave viral predictors with the seasonal components removed. We then fit the main models (described in the manuscript) but substituted the seasonally-adjusted viral variables for viral incidence.

Using the seasonally-adjusted incidence as a predictor, there is not a natural way to calculate the attributable percent. The exponentiated parameter estimates (e^β^) have the following interpretation: for every 1-fold increase of RSV above its seasonal average, pneumococcal pneumonia increases by e^β^-fold (e.g. if RSV increases 2-fold above the seasonal baseline, pneumococcal pneumonia incidence increases by 2*e^β^-fold). We compared estimates of attributable percent from the model that included the raw RSV incidence with the incidence rate ratio estimates from the models that included seasonally-adjusted RSV incidence (**Figure S3**). The age patterns estimated from the two models were consistent, with the strongest effect for the 0-2 month old children and the weakest effect for the 1-2 year olds. For all age groups, the models containing the raw unadjusted viral variables fit better than models containing the seasonally-adjusted viral variables (**Table S1).**

***Sensitivity of results to model selection.***

We considered whether the number of variables included in the model would impact the estimates of the attributable percent, including the number of terms representing harmonics, and temporal and geographic trends. We tested 18 different candidate models using BIC scores (**Table S1)**. All of the candidate models included an influenza and RSV variable, sine and cosine terms with 12-month periods, a dummy variable for state, and a dummy variable for vaccination period. We also tested models that had an added 6-month harmonic, and interactions terms that allowed each of the variables listed above to vary by state. Each of these candidate models was fit using either raw viral incidence or seasonally-adjusted viral incidence. The BIC score from each model was used to calculate a model weight (Symonds and Moussalli, Behav Ecol Sociobiol 2011, 65:13-21). In all instances, there was one model that had a significantly superior fit (**Table S1**). None of the best-fit models included interaction terms by state, indicating that viral effects were similar across states. The choice of model did not qualitatively alter the estimates of the percent of pneumococcal pneumonia attributable to RSV (**Figure S4).**

***Testing the association between pneumococcal disease and influenza and RSV variables with different lags***

We considered whether the association between pneumococcal disease was strongest with no lag, with lags of 1 to 4 weeks (with RSV or influenza occurring earlier than pneumococcal disease) or with pneumococcal disease preceding virus activity (a negative control). We compared the models using BIC criteria (using only observations that were present for all lagged variables) and calculated the attributable percent for each. Among the 0-1 year olds, the best models (by BIC) had the RSV variable unlagged, and lagging flu between 0 and 4 weeks had no impact on model fit (BIC change of <2). Lagging the RSV variables resulted in significantly poorer fit to the data. The models tested included the variables shown in **Table S1**, with the BIC score for the 0-11 month olds shown in parentheses. The estimates of the attributable percent for each model are shown in **(Figure S5).**
